# FcγRIIb Expression Is Decreased on Naive and Marginal Zone-Like B Cells From Females With Multiple Sclerosis

**DOI:** 10.3389/fimmu.2020.614492

**Published:** 2021-01-11

**Authors:** Stephanie Trend, Jonatan Leffler, Ingrid Teige, Björn Frendéus, Allan G. Kermode, Martyn A. French, Prue H. Hart

**Affiliations:** ^1^ Inflammation Laboratory, Telethon Kids Institute, University of Western Australia, Perth, WA, Australia; ^2^ Perron Institute for Neurological and Translational Science, University of Western Australia, Perth, WA, Australia; ^3^ Demyelinating Diseases Research Group, BioInvent International AB, Lund, Sweden; ^4^ Institute for Immunology and Infectious Disease, Murdoch University, Perth, WA, Australia; ^5^ Medical School and School of Biomedical Sciences, University of Western Australia, Perth, WA, Australia

**Keywords:** multiple sclerosis, B cells, FcγRIIb, females, Epstein-Barr virus, immune regulation

## Abstract

B cells are critical to the development of multiple sclerosis (MS), but the mechanisms by which they contribute to the disease are poorly defined. We hypothesised that the expression of CD32b (FcγRIIb), a receptor for the Fc region of IgG with inhibitory activities in B cells, is lower on B cell subsets from people with clinically isolated syndrome (CIS) or MS. CD32b expression was highest on post-naive IgM^+^ B cell subsets in healthy controls. For females with MS or CIS, significantly lower CD32b expression was identified on IgM^+^ B cell subsets, including naive and IgM^hi^ MZ-like B cells, when compared with control females. Lower CD32b expression on these B cell subsets was associated with detectable anti-Epstein Barr Virus viral capsid antigen IgM antibodies, and higher serum levels of B cell activating factor. To investigate the effects of lower CD32b expression, B cells were polyclonally activated in the presence of IgG immune complexes, with or without a CD32b blocking antibody, and the expression of TNF and IL-10 in B cell subsets was assessed. The reduction of TNF but not IL-10 expression in controls mediated by IgG immune complexes was reversed by CD32b blockade in naive and IgM^hi^ MZ-like B cells only. However, no consequence of lower CD32b expression on these cells from females with CIS or MS was detected. Our findings highlight a potential role for naive and marginal zone-like B cells in the immunopathogenesis of MS in females, which requires further investigation.

## Introduction

Multiple sclerosis (MS) is an immune-mediated disease of the central nervous system (CNS) characterised by episodes of inflammation that result in demyelination. A number of risk factors for MS have been identified (1), but the specific mechanisms of immune-mediated demyelination require further investigation (2). In and around MS lesions, there is evidence of infiltrating immune cells, an inflammatory cytokine milieu and deposits of immunoglobulins (Ig) and complement (3–5). Despite many attempts to characterise the Ig found in and around MS lesions and in cerebrospinal fluid, a common autoantigen target in MS has not been identified, suggesting that understanding autoantigen-independent Ig production and/or B cell responses may be a more pragmatic way to assess the associations of Ig with MS pathology.

Therapies that deplete B cells or prevent their entry to the CNS have improved clinical outcomes for people with relapsing-remitting (RR)MS, which is attributed to effects on peripheral memory B cells (MBC) (6). MBC are often defined as CD27^+^ B cells, but these are in fact a heterogeneous population that include marginal zone (MZ)-like B cells (IgM^+^IgD^+^), IgM-only MBC (IgM^+^IgD^−^), and class switched MBC (IgD^−^/IgG^+/−^/IgA^+/−^/IgE^+/−^). Each may exhibit diverse functional activities, in part due to differential expression of molecules such as CD21 and T_bet_ (7–9), independent of their B cell receptor (BCR) isotype. Moreover, CD27^−^ B cells can also be antigen experienced, including IgD^−^CD27^−^ cells, the so-called double negative (DN) B cells, which may be IgM^+^ or IgM^-^ (10). Given the importance of B cells for innate responses to novel pathogens and for contributing to serological memory through differentiation of MBC into plasma cells, total depletion of B cells may leave individuals with MS receiving anti-CD20 therapy vulnerable to infections (11, 12). Thus, the complexity of the B cell compartment in MS warrants further detailed examination with a view to developing more targeted therapies that do not result in global B cell losses.

The majority of risk associated with developing MS is non-heritable. A history of acute infectious mononucleosis and higher levels of serum antibodies against Epstein-Barr virus (EBV), a herpesvirus that chronically infects human IgD^+^CD27^+^ and IgD^−^CD27^+^ B cells (13), both contribute to a higher risk of developing MS (14). In addition, females have a 2.5 times higher risk of developing MS, presumably due to either the differential impact of sex hormones on immune cells (15), or the biallelic expression of immune response genes located on the X chromosome (16, 17). The increased risk of developing MS after viral infections (18–20) suggests that immune responses may interact with genetic risk factors in susceptible individuals to contribute to the development of MS.

Evidence of uninhibited B cell replication (autoproliferation), as well as “unswitched” and “memory” B cell phenotypes driving autoproliferation of T cells in cultured cells from people with MS (21) suggests that inappropriate B cell regulation may contribute to the disease phenotype. One regulatory receptor on B cells is the inhibitory receptor for immunoglobulin G (IgG), FcγRIIb (CD32b). CD32b downregulates BCR signals following engagement by IgG immune complexes (IgG-IC) *via* intracellular immunoreceptor tyrosine-based inhibition motifs (ITIMs) [reviewed in (22)], likely to prevent excessive B cell activation when a successful IgG response to a pathogen has been established. Furthermore, CD32b cross-linking by IgG-IC inhibits NF-κB signalling in B cells activated *via* Toll-like receptors (TLRs) (23, 24). Given that decreased CD32b expression on B cells is a feature of several autoimmune conditions (25–27) and plays an important role in maintaining peripheral B cell tolerance (28), we hypothesised that CD32b expression on B cell subsets from people with recently diagnosed MS or clinically isolated syndrome (CIS; pre-MS) would be lower than levels on B cells from healthy controls, and also examined the effect of CD32b engagement on cytokine expression following antigen-independent stimulation in the presence of IgG-IC.

Here, we report lower CD32b expression on total B cells, as well as naive and IgM^hi^ MZ-like B cell subsets in females with CIS or MS. Correlates of B cell CD32b expression were sought with markers previously measured in the serum of CIS or MS females. A functional assay based on polyclonal activation of B cells by a TLR7 ligand was developed to measure CD32b activity. We found that in naive and IgM^hi^ MZ-like B cells, TLR7-induced TNF expression was inhibited by CD32b engagement. However, the lower expression of CD32b observed on naive and IgM^hi^ MZ-like B cells of females with CIS or MS was not associated with decreased regulatory effects of CD32b engagement on cytokine expression in these cells.

## Methods

### Participants

Thirteen patients with CIS were recruited as part of the PhoCIS trial as previously described, with extensive phenotypic analyses previously performed (29–33). One additional patient with CIS and eight patients with MS were recruited at diagnosis of a symptomatic demyelinating event, as previously described (33). CIS or MS patient samples were collected a median of 12 days after their diagnostic magnetic resonance imaging (MRI) scan was performed, and 6/8 (75%) participants with MS were newly diagnosed at the time of blood sampling. Age- and sex-matched controls with no history of autoimmunity or current symptoms of acute infections were recruited. None of the individuals had been treated with MS-specific disease modifying therapies or corticosteroid therapy within 30 days. The cohorts were similar in age and sex between controls and patient groups (**Table 1**).

**Table 1:** Characteristics of the controls and patients with clinically isolated syndrome (CIS) or multiple sclerosis (MS) included in the dataset.

	Controls (n = 16)	CIS or MS (n = 22)	p-value
Female sex [n, (%)]	9 (56%)	13 (59%)	0.86
Age [median, (IQR)]	41.5 (33.0–47.6)	37.3 (33.7–45.2)	0.53

Categorical comparisons were made using a Chi-squared test. Two group comparisons between control and patients with CIS or MS were tested using a Mann-Whitney test.

Of the CIS patients, 11/14 converted to MS within 1 year of sample collection (79%), and 1/14 within 2 years of sample collection. The characteristics of the CIS and MS groups were not significantly different in their age, sex and CD32b expression on B cell subsets (**Supplementary Table 1; Supplementary Figure 1**). Therefore, patients with CIS or MS were studied as a group and compared with controls.

### Sample Collection

Peripheral venous blood was collected from patients with CIS or MS into sodium heparin and SST vacutainers (BD) to isolate peripheral blood mononuclear cells (PBMC) and serum, respectively. Controls were recruited in a separate study and only PBMC were isolated. This study was reviewed and approved by the Sir Charles Gairdner Hospital Human Research Ethics committee (2006–073) and Bellberry Human Research Ethics Committee (2014-02-083). All participants provided their written informed consent to participate in this study. This research was carried out in accordance with the recommendations of the National Health and Medical Research Council of Australia’s National Statement on Ethical Conduct in Human Research.

### Measurement of Serum Immunoglobulins, B-Cell Activating Factor, and Epstein-Barr Virus Antibodies

Total IgG, IgG_2–4_, IgM, and IgA were measured in serum from people with CIS or MS as previously described, using bead-based commercial immunoassays (33). Serum B cell activating factor (BAFF) was measured using an enzyme-linked immunoassay according to the manufacturer’s instructions (R&D Systems; Minneapolis, United States of America). Serum levels of antibodies against EBV viral capsid antigen (VCA; IgM and IgG) and EBV nuclear antigen (EBNA)-1 (IgG) were assessed by PathWest diagnostic laboratory (Perth, Western Australia), as previously described (33).

### Generation of IgG Immune Complexes (IgG-IC)

Heat-aggregated IgG was used to model the effects of IgG-IC in experiments. Heat-aggregated IgG-IC were generated by heating human serum-derived IgG (Sigma-Aldrich, St. Louis, USA) at 63°C for 20 min before placing on ice. Insoluble IgG-IC were sedimented by centrifugation at 13,000 x g for 10 min as previously described (34), and the concentration of insoluble IgG-IC was determined by subtracting the amount of soluble IgG detected in the supernatant from the starting concentration of total IgG (measured using a NanoDrop 2000 spectrophotometer; Thermo Scientific, Wilmington, USA).

### Peripheral Blood Mononuclear Cell (PBMC) Isolation and Thawing

PBMC were isolated using Lymphoprep (Axis-Shield, Dundee, UK) and cryopreserved in liquid nitrogen in 10% dimethyl sulfoxide (DMSO) in fetal bovine serum (FBS; HyClone) until utilisation. For experimental assays, cryopreserved PBMCs were thawed, washed and resuspended in RPMI 1640-based (Gibco; Thermo Fisher Scientific, Waltham, USA) thawing medium containing 10% FBS as previously described (30). One million cells from each sample were washed twice in RPMI and utilised immediately for *ex vivo* surface staining to identify PBMC subsets and quantify their expression of CD32b. Remaining cells were cultured with indicated stimuli to investigate intracellular cytokine responses in B cell subsets, as described below.

### PBMC Phenotyping

Monoclonal antibodies generated in mice against CD19 (BUV737 clone SJ25C1), CD20 (BUV737 clone 2H7), CD24 (BV786 clone ML5), CD27 (BB700 clone L128), CD38 (BV510 clone HIT2), CD64 (FcγRI; BV711 clone 10.1), IgM (BUV395 clone G20-127) and IgD (PE-CF594 clone IA6-2) were obtained from BD Biosciences (Franklin Lakes, USA). Anti-CD32b (conjugated to AlexaFluor488) was generated by BioInvent, Sweden (35). Thawed PBMC were washed in PBS and then incubated with live/dead stain (FVS575V; BD Biosciences) for 30 min at 4°C according to the manufacturer’s recommendation. Cells were then washed twice with flow cytometry buffer (4% FBS in PBS) and incubated with a cocktail of antibodies against surface markers containing brilliant stain buffer (BD Biosciences) for 30 min at 4°C. Following incubation, cells were washed twice with flow cytometry buffer, fixed for 10 min at room temperature using a solution of 2% paraformaldehyde, washed twice and then resuspended in flow cytometry buffer for storage at 4°C until analysis.

### PBMC Stimulation

R848 (Resiquimod) was selected as a polyclonal B cell activator, which mediates its effects *via* TLR7 and TLR8 [the latter not expressed on B cells (36)]. PBMC were cultured in 96-well U-bottom polypropylene plates at 8 × 10^5^ cells per well in RPMI 1640 medium supplemented with 10% FBS, 5 µg/ml gentamicin, 2 mM l-glutamine, 50 µM 2-β-mercaptoethanol (all Sigma-Aldrich) (29), as well as 1 µL/ml GolgiPlug (BD Biosciences) (37) in order to maximise detection of intracellular IL-10 and TNF, and to inhibit the effects on B cells of products from activated bystander cells (38). Where indicated, cell culture medium was supplemented with IgG-IC (100 µg/ml) and/or R848 (500 ng/ml; InvivoGen; San Diego, USA); these concentrations were optimised in preliminary experiments, which also determined that inclusion of GolgiPlug did not substantially alter cell viability (not shown).

To investigate the role of CD32b in IgG-IC-mediated cell responses, the PBMCs were pre-incubated at 37°C for 30 min with a fully human specific anti-CD32b antagonistic antibody (BI-1206; hIgG1L-CD32b-006-G11) (39) at 10 µg/ml prior to cell stimulation with IgG-IC and R848. The specificity of the antagonistic antibody for CD32b was previously confirmed (35), and substantial reduction in the detection of CD32b in B cells by flow cytometry in the presence of this antibody was confirmed in our laboratory (not shown).

Following pre-incubation and addition of stimuli, cell suspensions were incubated for 18 h at 37°C in 5% CO_2_. Cells were then washed with cold PBS, incubated with live/dead stain followed by antibodies as described above. Following surface staining, cells were fixed, permeabilised and washed using a Cytofix/Cytoperm kit (BD Biosciences), then incubated for 30 min at 4°C with antibodies obtained from BD Biosciences against TNF (PE-Cy7 clone MAb11) and IL-10 (PE clone JES3-19F1). Following incubation, cells were washed and resuspended in flow cytometry buffer and stored at 4°C until analysis.

### Flow Cytometry

PBMC stained with antibodies were analysed using flow cytometry. Data were collected using an LSR Fortessa (BD Biosciences). In order to ensure consistency between median fluorescence intensity (MFI) values obtained in separate experimental batches, Ultra Rainbow calibration beads (Spherotech, Lake Forest, USA) were used to adjust photomultiplier voltages of fluorophore channels to target MFI values for bead peaks. Approximately 150,000 PBMC were acquired for each sample, and data analysis and compensation were carried out using FlowJo software (v10.6.1).

**Figure 1:**
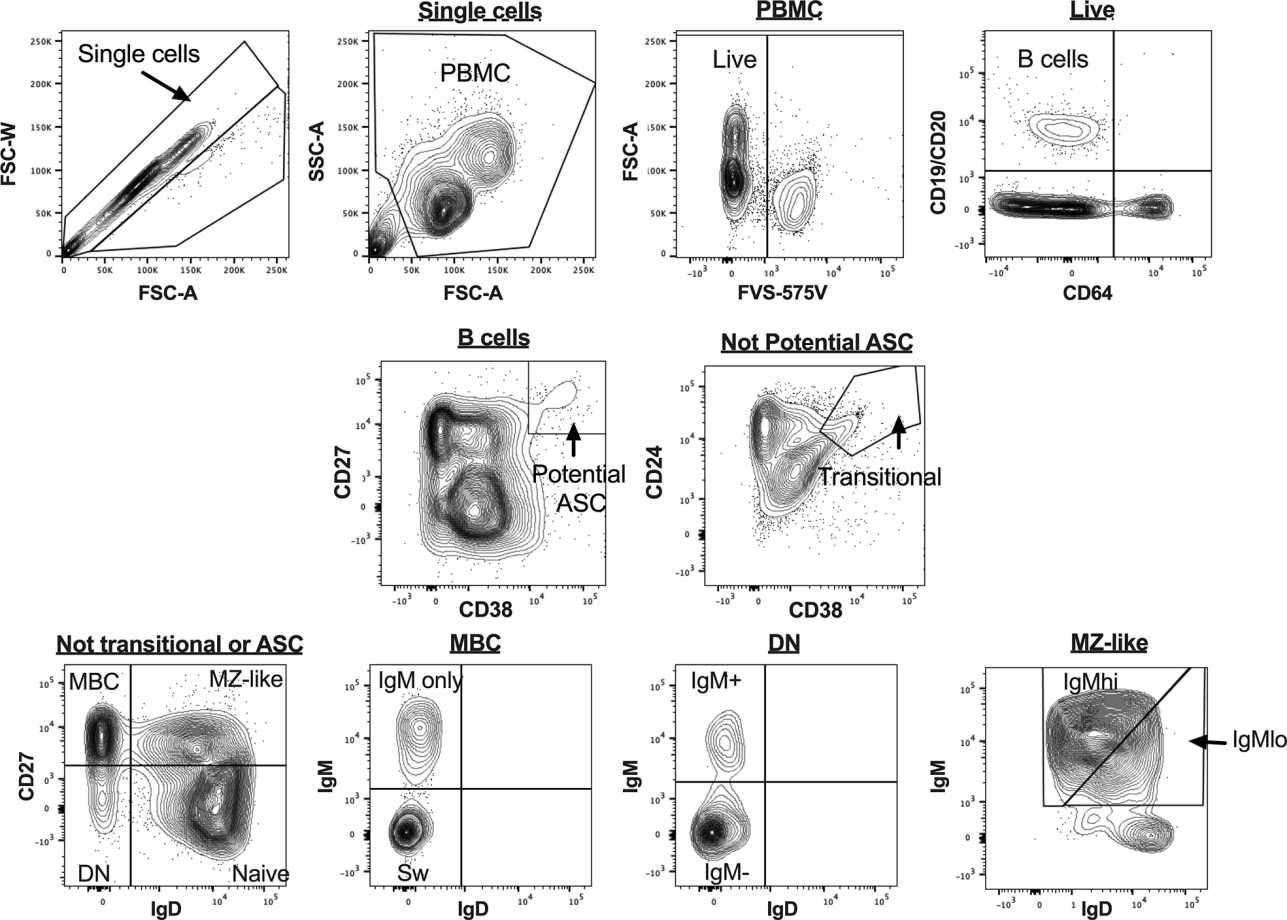
B cell gating strategy for analysis of uncultured peripheral blood mononuclear cells (PBMC) for flow cytometry data. Figures are taken from a representative sample. ASC, antibody secreting cells; MBC, memory B cells; MZ-like, marginal zone-like B cells; DN, double negative B cells; Sw MBC, class switched MBC.

**Figure 2:**
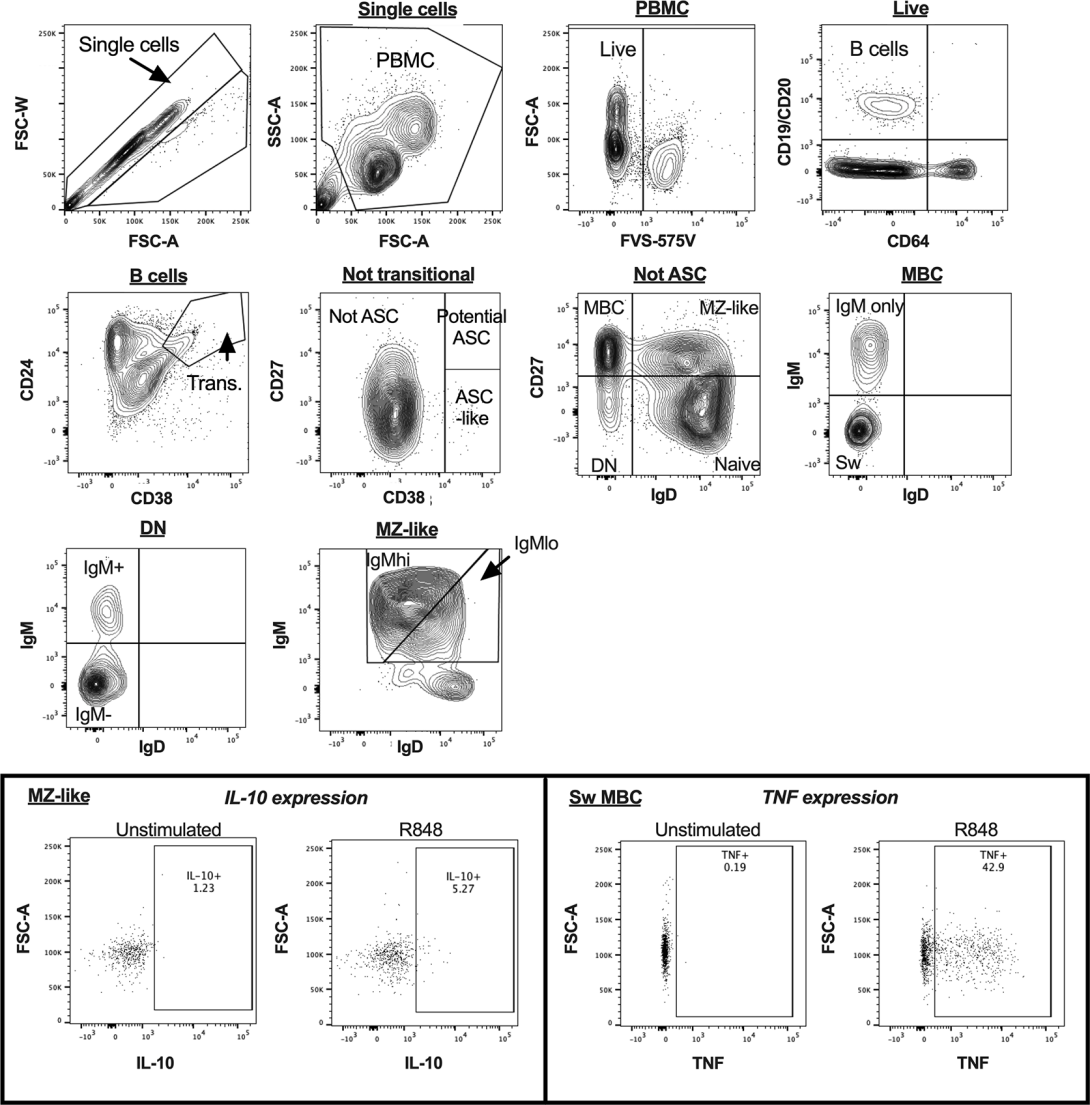
B cell gating strategy for analysis of cultured peripheral blood mononuclear cells (PBMC) in a representative sample by flow cytometry. The inset panels show the gating used to identify cytokine-expressing cells for interleukin 10 (IL-10) in MZ-like cells, and tumour necrosis factor (TNF) in class-switched MBC, with figures illustrating both unstimulated and R848-stimulated cells for each cytokine. ASC, antibody secreting cells; MBC, memory B cells; MZ-like, marginal zone-like B cells; DN, double negative B cells; Sw MBC, class switched MBC.

B cell subsets were identified in uncultured PBMCs as shown in **Figure 1**, as CD19^+^ and/or CD20^+^ cells not expressing CD64. Plasmablasts (PB) and antibody-secreting cell (ASC)-like cells were excluded from analyses, as shown in **Figures 1** and **2**, respectively. MZ-like B cells (CD27^+^IgD^+^IgM^+^) were divided into IgM^hi^ and IgM^lo^ MZ-like B cells as recently described (7). CD32b expression on all B cell subsets was examined using MFI. Intracellular cytokines IL-10 and TNF were examined in stimulated and unstimulated cells cultured overnight, as shown in **Figure 2** (inset). Intracellular cytokine expression in stimulated B cells was measured as % cytokine positive cells.

### Statistical Analyses

Shapiro-Wilk normality tests were used to confirm that expression of CD32b and B cell frequencies were not normally distributed. Therefore, continuous data from controls and patients with CIS or MS were compared using Mann-Whitney tests. Categorical data were compared using a Chi-squared test. The differences between CD32b expression in B cell subsets within controls was compared using a Friedman test, and post-test comparisons between B cell subsets performed using the two-stage linear step-up procedure of Benjamini, Krieger and Yekutieli (40). Correlations between CD32b expression and other serum factors were analysed used Spearman’s rho.

Cell culture data were normally distributed according to Shapiro-Wilk normality tests and therefore parametric statistical tests were utilised in their analyses. Cytokine-expressing cell frequencies were compared within individuals under different culture conditions using paired t-tests. Frequencies of TNF^+^ cells in naive B cells and IgM^hi^ MZ-like B cells were compared between control females and females with CIS or MS in R848- and IgG-IC+R848-stimulated cells using 2-way repeated measures ANOVA. In all analyses, a p-value<0.05 was considered statistically significant.

Statistical analyses were performed using Prism (v8, GraphPad, San Diego, CA, USA) and SPSS (v25, IBM, Armonk, USA) and figures were generated using R software (version 1.1.463, package ggplot) (41) and Prism.

## Results

### CD32b Expression Is Lower on the Naive and IgM^hi^ MZ-Like B Cells of Females With CIS or MS

**Figure 3:**
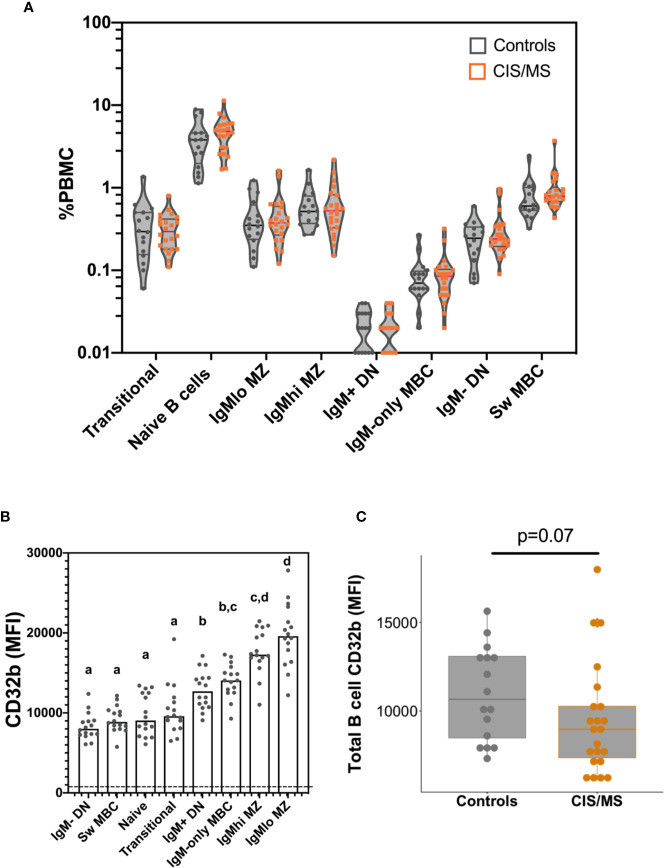
B cell frequencies and CD32b expression on B cells isolated from the blood of the control and CIS or MS patient cohorts. **(A)** Frequencies of B cell subsets as a proportion of total PBMC. Violin plots show the range and quartiles of B cell subset frequencies in controls (n = 16; grey), or patients with CIS or MS (n = 22, orange). **(B)** Relative expression of CD32b on B cell subsets from controls. Bars show median expression with a dot shown for each individual (n = 16). The dashed line indicates the approximate value of background CD32b staining, which was measured using a fluorescence minus one stain on total B cells (gated in **Figure 1**). The letters above data indicate B cell subsets that were not significantly different to one another in their CD32b expression (n = 16), calculated using a Friedman test, with the results of a multiple comparison post-test shown using the two-stage linear step up procedure of Benjamini, Kreiger and Yekutieli. **(C)** CD32b expression on total B cells from controls and patients with CIS or MS. CD32b expression is shown using Tukey’s boxplots displaying median values and inter-quartile ranges within boxes, with a dot shown for each individual; potential significance was determined using Mann-Whitney tests.

There were no significant differences between controls and patients with CIS or MS in total B cell frequencies (not shown), or in any B cell subset frequencies (%PBMC; **Figure 3A**).

To better understand the role of CD32b in the function of each different B cell subset, the pattern of CD32b expression was initially examined in healthy controls. All B cell populations expressed CD32b (**Figure 3B**). However, the highest level of expression of CD32b was detected in CD27^+^ B cell subsets that expressed IgM, including IgM^lo^ MZ-like B cells, IgM^hi^ MZ-like B cells, IgM-only MBC and IgM^+^ DN B cells (**Figure 3B**). Transitional, naive and IgM^-^ B cell subsets expressed relatively lower levels of CD32b (**Figure 3B**).

**Figure 4:**
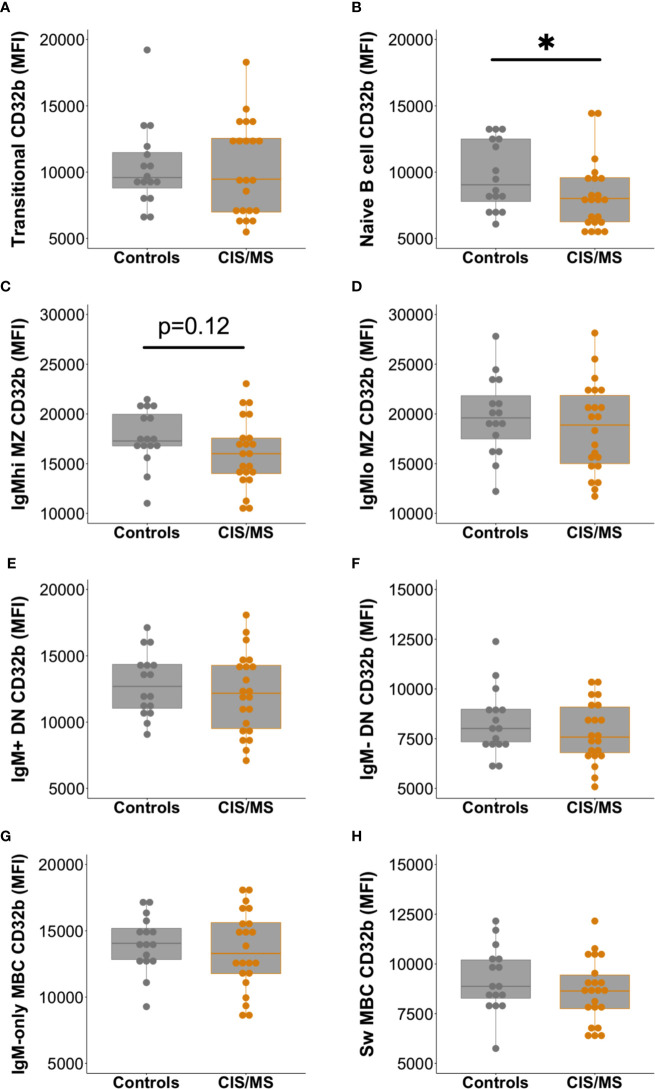
Expression of CD32b on B cell subsets from controls (n = 16, grey circles) compared with patients with CIS or MS (n = 22, orange circles). CD32b expression is shown using Tukey’s boxplots on **(A)** transitional B cells; **(B)** naive B cells; **(C)** IgM^hi^ MZ-like B cells; **(D)** IgM^lo^ MZ B cells; **(E)** IgM^+^ DN B cells; **(F)** IgM^-^ DN B cells; **(G)** IgM-only MBC; and **(H)** class switched (Sw) MBC. A dot is shown for each individual. Mann-Whitney tests were used to compare groups; *p-value<0.05.

Expression of CD32b was next assessed on total B cells from people with MS or CIS and compared with controls. There was a clear trend towards decreased expression of CD32b in patients with CIS or MS compared with controls on total B cells (**Figure 3C**), though this difference was not statistically significant (p = 0.07). Assessing individual B cell subsets, expression of CD32b was significantly lower on naive B cells in patients with CIS or MS, compared with controls (**Figure 4B**). No other comparisons of CD32b expression in B cell subsets were statistically significant (**Figure 4** and not shown).

**Figure 5:**
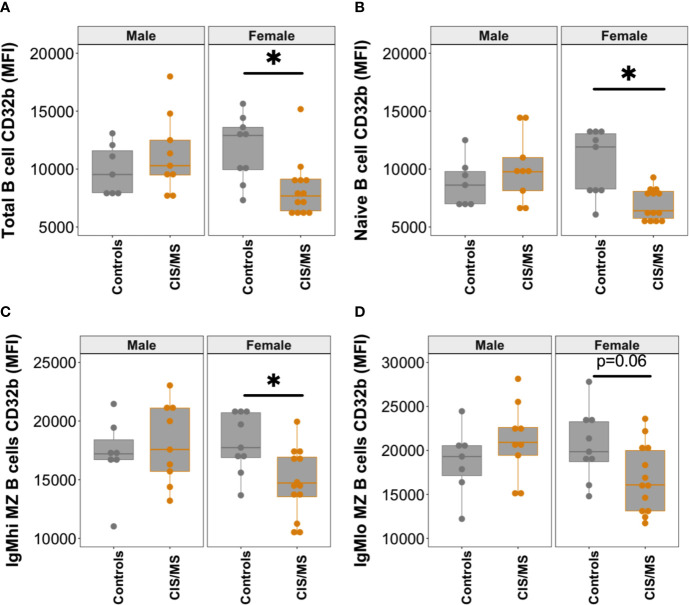
CD32b expression on B cell subsets from males and females with CIS or MS compared with controls. CD32b expression is shown using Tukey’s boxplots on **(A)** total B cells; **(B)** naive B cells; **(C)** IgM^hi^ MZ B cells; and **(D)** IgM^lo^ MZ B cells from healthy controls (n = 7 males, 9 females; grey circles), and patients with CIS or MS (n = 9 males and 13 females; orange circles). A dot is shown for each individual. Mann-Whitney tests were used to compare groups; *p-value<0.05.

Given the higher prevalence of MS in females, the cohort was separated and examined by sex to assess whether there was any difference between males and females in CD32b expression. Significantly lower expression of CD32b was detected on total B cells, naive B cells and IgM^hi^ MZ-like B cells from females with CIS or MS compared with control females (**Figures 5A–C**). A trend towards lower CD32b expression on IgM^lo^ MZ-like B cells from female patients was observed but the difference was not statistically significant (p = 0.06, **Figure 5D**). There were no differences in CD32b expression levels on B cells from males with CIS or MS and controls. Together, these results demonstrated that IgM^+^ B cells from females but not males with CIS or MS expressed lower levels of CD32b compared with female controls.

### Decreased CD32b Expression on Naive and MZ-Like B Cells Was Associated With Higher Serum BAFF Levels and EBV VCA IgM Seropositivity

Since significantly lower CD32b expression on total, naive and IgM^hi^ MZ-like B cells and non-significantly lower expression on IgM^lo^ MZ-like B cells from females with CIS or MS was detected, CD32b expression on these, and other B cell subsets was examined for associations with serum factors that may be related to B cell dysfunction. Variables investigated included serum levels of BAFF, which are increased in people with MS (42), and anti-EBV antibodies, since EBV has been implicated in the pathogenesis of MS (14). In addition, total non-specific IgM levels were compared with CD32b expression levels on cells, since MZ-like B cells are the main producers of serum IgM (43). Serum levels of other Ig, including IgA, total IgG and IgG subclasses were also included for comparison. No serum was available for controls.

**Figure 6:**
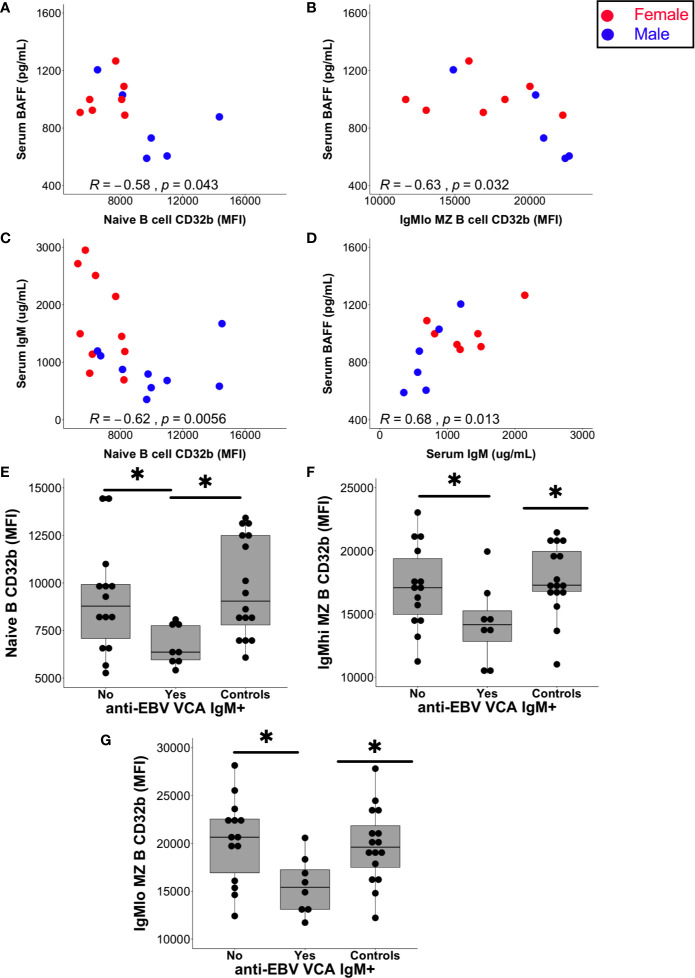
Relationships between serum factors and B cell subset CD32b expression. **(A)** Correlation between naive B cell CD32b expression (MFI) or **(B)** IgM^lo^ MZ B cell CD32b expression with serum levels of BAFF (**A, B**; n = 13). **(C)** Correlation between naive B cell CD32b expression and serum IgM levels (n = 19). **(D)** Correlation between serum IgM and BAFF levels (n = 13). Values shown were calculated for males and females combined using Spearman’s rho; results from males are shown as blue circles and results from females are shown as red circles; p values<0.05 were considered statistically significant. **(E)** Levels of naive B cell CD32b expression, **(F)** IgM^hi^ MZ-like B cell CD32b expression, and **(G)** IgM^lo^ MZ B cell CD32b expression, according to anti-EBV VCA IgM antibody detection status (Yes, n = 8; No, n = 14; Controls, n = 16). For control samples, no serum was available for EBV serology testing. Data in **(E–G)** are presented using Tukey’s boxplots with a dot shown for each individual. Potential differences in CD32b expression between groups were tested using Mann-Whitney tests; *p<0.05.

Serum BAFF levels in patients with CIS or MS negatively correlated with the expression of CD32b on naive B cells (p = 0.043; **Figure 6A**) and IgM^lo^ MZ-like B cells (p = 0.02; **Figure 6B**), but not with other B cell subsets. Females with CIS or MS had significantly higher levels of serum IgM compared with males, and although females had higher median BAFF levels in serum than males, this was not statistically significant (**Supplementary Figure 2A**). While no relationship was detected between serum IgM levels and CD32b expression on other B cell subsets, serum IgM levels correlated negatively with CD32b expression on naive B cells (**Figure 6C**). Positive correlation between serum IgM and BAFF levels was also detected (**Figure 6D**). Serum levels of total IgG, IgG_2_, IgG_3_ and IgG_4_, as well as IgA, did not correlate with any B cell subset CD32b expression or serum BAFF levels (not shown).

The serum of all individuals with CIS or MS contained anti-EBV EBNA-1 IgG antibodies at a titre of 1:32 or greater indicating chronic EBV infection, as expected (44). In addition, the serum of 8/22 (36%) people with CIS or MS also contained anti-EBV VCA IgM antibodies, which is considerably higher than the 3–6% rate of seropositivity demonstrated in other clinical settings (45). Furthermore, anti-EBV VCA IgM antibodies were detectable in the sera of six females with CIS, one male with CIS and one female with MS. The increased detection of anti-EBV VCA IgM in females compared with males in the CIS or MS patient group was borderline significant (**Supplementary Figure 2B**). Analysis of people with CIS or MS separated into groups of those with and without detectable anti-EBV VCA IgM antibodies demonstrated that CD32b expression was significantly lower on naive, IgM^hi^ and IgM^lo^ MZ-like B cells in EBV VCA IgM seropositive compared with seronegative individuals (**Figures 6E–G**). Median levels of serum BAFF and total IgM levels were non-significantly higher in those with detectable anti-EBV VCA IgM (**Supplementary Figures 2C, D**), but other serum total Ig levels and IgG subclass proportions (%IgG) were similar between anti-EBV VCA IgM seropositive and seronegative individuals. Anti-EBV VCA IgM and total IgM were not correlated with one another (not shown).

Taken together, these data from patients with CIS or MS suggest that lower CD32b expression on naive and MZ-like B cell subsets may be associated with higher serum levels of BAFF and IgM, and EBV VCA IgM antibody seropositivity, and this phenotype is predominantly observed in females.

### Effect of CD32b Engagement on Inhibition of TLR7-Induced Cytokine Production in B Cell Subsets

Since naive and MZ-like B cells were the only B cell subpopulations exhibiting decreased CD32b expression in females with CIS or MS when compared with healthy controls, and MZ-like B cells are important for innate immune responses, antigen-independent B cell stimulation was utilised to investigate potential differences in the functional effects of CD32b engagement in patients and controls. An 18 h stimulation experiment was established to investigate the capacity of IgG-IC to modulate B cells activated by TLR7 ligation. PBMC were incubated with R848 (a TLR7 agonist) alone or in addition to IgG-IC, and intracellular cytokine expression was examined.

**Figure 7:**
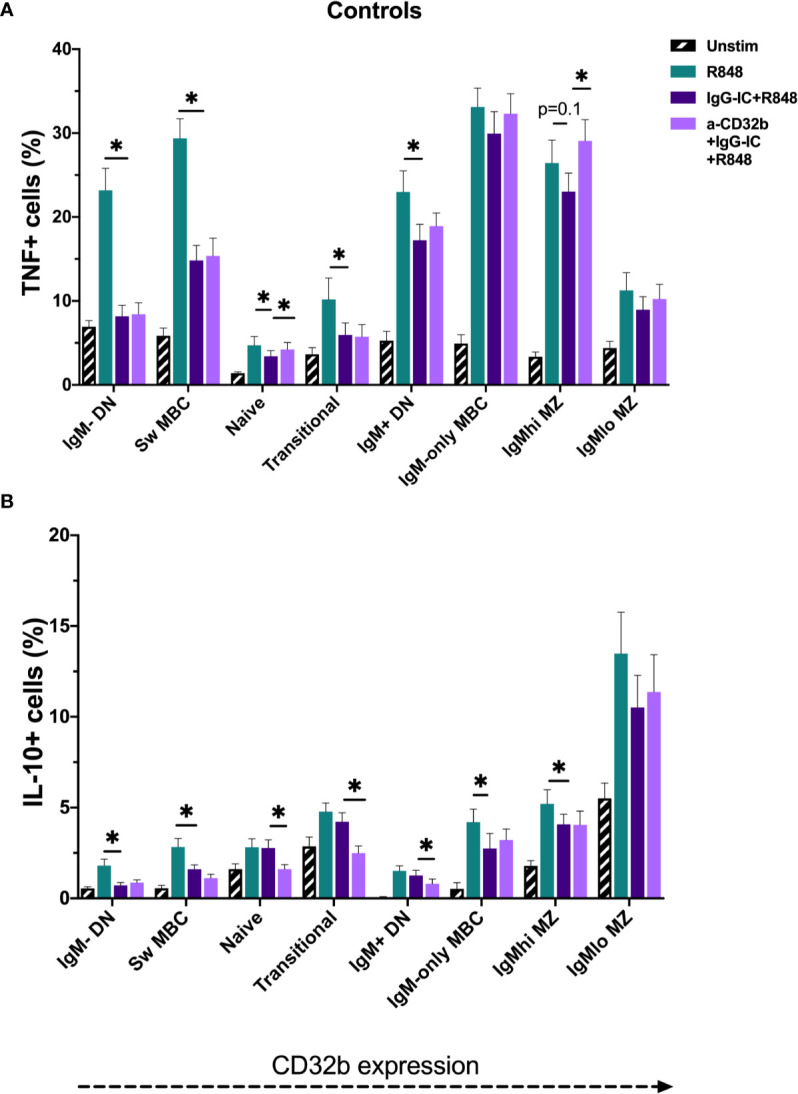
TNF and IL-10 produced by B cell subsets from the controls cultured for 18 h with Resiquimod (R848) and IgG immune complexes (IgG-IC). The data show **(A)** TNF^+^ cell frequencies, and **(B)** IL-10^+^ cell frequencies. Unstimulated cells in culture are shown as black striped bars. R848-stimulated cells (green bars) were compared with IgG-IC+R848 (dark purple bars), and IgG-IC+R848 stimulated cells were compared with cells cultured with anti-CD32b antagonist (a-CD32b)+IgG-IC+R848 (light purple bars). Bars show mean ± SEM. *p<0.05 in paired t-tests.

In unstimulated cultures, cells from CIS or MS patients contained higher frequencies of TNF^+^ IgM^hi^ MZ-like B cells compared with controls (median 3.9% vs 2.3% TNF+ cells; p = 0.048). However, when examining data from only females, no difference in IgM^hi^ MZ-like B cells TNF^+^ cell frequency between the groups was observed. No differences in the frequencies of TNF^+^ or IL-10^+^ B cells were detected in other unstimulated cultures. Culture with R848 stimulated expression of TNF and IL-10 in all B cell subsets (**Figures 7A, B**).

To investigate the effects of CD32b engagement by IgG-IC on cytokine expression in different B cell subsets stimulated with R848, cells from controls were first studied. The addition of IgG-IC+R848 decreased R848-induced TNF^+^ and IL-10^+^ cell frequencies in most R848-stimulated B cell populations; this included both low and high CD32b-expressing B cell subsets (**Figures 7A, B**). To determine the contribution of CD32b to the inhibitory effect of IgG-IC on R848-induced TNF or IL-10 responses in B cell subsets, a specific CD32b antagonist antibody was used to block CD32b activity, and the differences in cytokine responses in IgG-IC+R848- and anti-CD32b+IgG-IC+R848-stimulated cells were examined. In control cells, addition of anti-CD32b significantly increased the frequency of TNF^+^ B cells in naive and IgM^hi^ MZ-like B cells (**Figure 7A**), suggesting that inhibition of TNF expression by IgG-IC was mediated by CD32b in these subsets. Some effects of anti-CD32b antibody were observed in other IgM+ B cells, but the differences were not statistically significant. In contrast to TNF^+^ B cells, blockade of CD32b further decreased the frequency of IL-10^+^ cells amongst naive, transitional and IgM^+^ DN B cells, but not other B cell subsets (**Figure 7B**). In summary, IgG-IC reduced both TNF and IL-10 expression in many B cell subsets. However, this effect was mediated through CD32b in naive and IgM^hi^ MZ-like B cells for TNF only, as the effect of IgG-IC was reversed by CD32b blockade. Since we determined that CD32b engagement did not inhibit IL-10 responses in B cells, only TNF responses were further examined.

**Figure 8:**
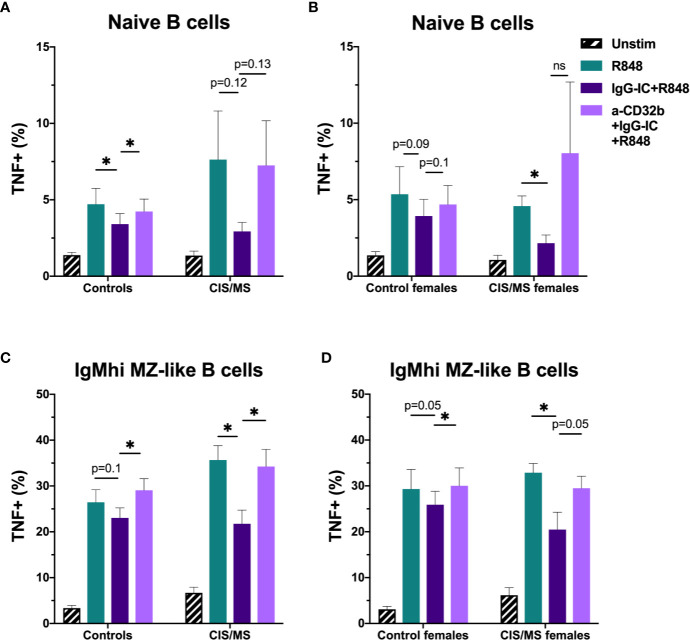
The effect of sex and CIS/MS on TNF produced by B cell subsets after culture for 18 h with Resiquimod (R848) and IgG immune complexes (IgG-IC). Data are shown for the total cohort **(A, C)** separated into controls (n = 16) or patients with CIS or MS (n = 22), or females **(B, D)**, separated into controls (n = 9) or patients with CIS or MS (n = 13). **(A)** TNF^+^ cell frequencies in naive B cells from all participants; **(B)** TNF^+^ cell frequencies in naive B cells from females; **(C)** TNF^+^ cell frequencies in IgM^hi^ MZ-like B cells from all participants; and **(D)** TNF^+^ cell frequencies in IgM^hi^ MZ-like B cells from females. Unstimulated cells in culture are shown as black striped bars. R848-stimulated cells (green bars) were compared with IgG-IC+R848 (dark purple bars), and IgG-IC+R848 stimulated cells were compared with cells cultured with anti-CD32b antagonist (a-CD32b)+IgG-IC+R848 (light purple bars). Bars show mean ± SEM. *p<0.05 in paired t-tests.

To assess whether there were disease- and sex-specific differences in the inhibitory capacity of CD32b engagement on TNF expression in CIS or MS patients, TNF data in B cell subsets with the clearest evidence of an inhibitory effect of CD32b engagement by IgG-IC in controls (i.e. naive and IgM^hi^ MZ-like B cells) were examined. In the total cohort and subsequently in females only, TNF expression was compared between controls and patients with CIS or MS (**Figure 8**). The results appeared similar whether data for the total cohort (**Figures 8A, C**) or females only were included in the analyses (**Figures 8B, D**). There were no significant differences in TNF^+^ cell frequencies in R848-stimulated cells from controls or patients, either using data from females only or the complete cohort (**Figure 8**). Naive B cell TNF^+^ cell frequencies in R848-stimulated cultures were regulated by CD32b in controls since anti-CD32b reversed the effect of IgG-IC on TNF expression (**Figure 8A**), and a similar trend was observed in female controls (**Figure 8B**). Naive B cell TNF^+^ cell frequencies were not significantly inhibited by IgG-IC in patients with CIS or MS despite exhibiting a similar pattern to controls in the data (**Figure 8A**). Although IgG-IC significantly inhibited TNF expression in naive B cells from females with CIS or MS, the effect of blocking CD32b in this group was not significant (**Figure 8B**). IgM^hi^ MZ-like B cell TNF^+^ cell frequencies in R848-stimulated cultures were regulated by CD32b engagement in total and female only controls, but the effects of IgG-IC on TNF^+^ cell frequencies were not significant (**Figures 8C, D**). In patients with CIS or MS, IgG-IC inhibited TNF^+^ cell frequencies regardless of sex (**Figures 8C, D**), and the effects of blocking CD32b were statistically significant in the analysis including both male and female patients (**Figure 8C**) and borderline significant in female patients with CIS or MS (**Figure 8D**). Finally, in repeated measures 2-way ANOVA analyses, TNF+ cell frequencies in R848- and IgG-IC+R848-stimulated naive or IgM^hi^ MZ-like B cells were not significantly different between controls and patients with CIS or MS, or control females and female patients with CIS or MS (not shown). This indicated that despite lower CD32b expression on these cell types in females with CIS or MS, regulation of TNF expression by IgG-IC was not different to controls in these patients.

Overall, in stimulated B cells from controls, IgG-IC decreased R848-induced TNF expression in a range of B cell subsets, but this was demonstrated to be a result of CD32b blockade only in naive and IgM^hi^ MZ-like B cells. When investigating the effects of CD32b engagement on B cell subsets from females with CIS or MS, naive and IgM^hi^ MZ-like B cell TNF expression appeared to be regulated by IgG-IC *via* CD32b, but the effect was not statistically significant. Despite this, lower CD32b expression was not associated with a decreased capacity to inhibit TNF expression in females with CIS or MS compared with female controls.

## Discussion

Signalling through CD32b (FcγRIIb) on B cells contributes to peripheral tolerance of self-reactive B cell clones and reduces “bystander” activation of unrelated clones during immune responses (28). Moreover, while CD32b expression is decreased on B cells in several autoimmune diseases, increasing the expression of CD32b on B cells in autoimmune mouse strains is associated with restoration of tolerance (46). Here we report for the first time that total B cells, and in particular naive B cells and IgM^hi^ MZ-like B cells from females with CIS or MS, express lower levels of CD32b compared with healthy control females. In addition, we demonstrated that lower CD32b expression was associated with higher serum BAFF and IgM levels, and EBV VCA IgM seropositivity. In a search to understand the biological significance of lower CD32b expression on these cells, an *in vitro* assay was developed to test the functional properties of CD32b. By investigating B cell subsets polyclonally activated by R848 and the inhibitory effects of IgG-IC alone or combined with a CD32b-blocking antibody, we determined that the suppressive effect of IgG-IC on TNF expression in naive and IgM^hi^ MZ-like B cells was mediated by CD32b in controls examined as a total group, or in IgM^hi^ MZ-like B cells in control females only. In females with CIS or MS, naive and IgM^hi^ MZ-like B cell inhibition mediated by IgG-IC was partially abrogated by blocking CD32b, though this effect was not statistically significant. However, lower expression of CD32b on naive and IgM^hi^ MZ-like B cells from females with CIS or MS was not associated with decreased effects of CD32b engagement and/or downstream signalling on TNF expression. A different functional assay involving an alternative pathway regulated by CD32b ligands may have demonstrated a measurable consequence of reduced B cell CD32b expression that could contribute to the pathogenesis of MS in females.

Our data suggest that CD32b plays a role in inhibiting IgM^+^ B cell activation for TNF production, particularly in IgM^hi^ MZ-like B cells and naive B cells, and that CD32b expression varies alongside relative IgM expression during B cell maturation. In agreement with our findings, Glass and colleagues recently reported a correlation between IgM and CD32b expression across B cell populations (47). Furthermore, CD32b is involved in inhibiting IgM^+^ B cell responses following BCR engagement by IgG_3_ in HIV^+^ individuals *via* IgM clustering on the cell surface (48). While we demonstrated that most B cell populations increased TNF^+^ cell frequencies in response to TLR7 ligation, and expression of TNF was inhibited by addition of IgG-IC, this inhibition was only clearly dependent on CD32b in naive and IgM^hi^ MZ-like B cells. Cytokine production by class-switched MBC and IgM^-^ DN B cells was strongly inhibited by IgG-IC, but CD32b blockade was without effect, suggesting that CD32b is less important in inhibiting activation of class-switched MBC, in line with the lower expression of this molecule on these cells. Alternative receptors for IgG Fc regions such as FcRL4 or FcRL5 may be more important in regulating these cell types with lower CD32b expression (49), and should be investigated in future. Furthermore, intracellular production of cytokines such as TNF reflects signalling downstream of CD32b, which may also differ between B cell subpopulations and should be examined. These results highlight the importance of including specific receptor antagonists to determine the roles of different IgG receptors on B cell functions.

Lower CD32b expression on B cell subsets from females with CIS or MS was detected in this study. Three previous studies of people with CIS or remitting relapsing MS (RRMS) had demonstrated no difference in expression of mRNA of the gene encoding CD32b in total leukocytes or CD32b expression on B cells from people with MS compared with healthy individuals (50–52). However, we utilised different methodology to examine B cell subsets, and most importantly, we have examined expression on cells from males and females separately, which may explain the differences between our study and others. Our results suggest that lower CD32b expression might contribute to the increased prevalence of MS observed in females; further research is required to examine this possibility. A previous investigation of CD32b expression in healthy patients demonstrated lower CD32b expression on total CD19+ B cells from females compared with males, though we found no such effect in controls in this study (53). However, the control participants in the previous study were substantially older than our cohort, and the authors reported that CD32b expression decreased with age in females, which may explain the differences between the two investigations. Despite this, it is possible that females downregulate CD32b to a larger extent when B cells are activated (e.g. in CIS/MS patients) because of a potential effect of biallelic expression of immune response genes located on the X chromosome that regulate the CD32b signalling pathway, such as *Btk* (24, 54), or because of the influence of female sex hormones on B cells (15). It is unclear whether genetic polymorphisms in our participants also contributed to the expression and/or activity of the CD32b receptor observed, as has been reported for systemic lupus erythematosus (55, 56). Though there is no known genetic risk of MS associated with FCGR2B gene polymorphisms, polymorphisms in the genes encoding FcγRIIa and FcγRIIIb in people with MS are linked to a more benign disease course (57). Despite the significantly lower CD32b expression on naive and IgM^hi^ MZ-like B cells from females with CIS or MS compared with controls, and the capacity of TNF expression in these cells to be regulated by IgG-IC through CD32b, a functional outcome dependent on reduced CD32b was not detected. In patients with systemic lupus erythematosus, lower CD32b expression on B cells is associated with increased calcium signalling in response to BCR engagement with anti-IgG compared to B cells with normal CD32b expression (27). As naive B cells are less responsive to T-independent signals than MZ-like B cells, an assay measuring BCR-mediated activation or cell proliferation might provide an additional strategy to test the role of CD32b in these cells. Moreover, alternative methods of CD32b engagement might be more informative, such as bead-bound IgG, which was not used in this study due to the technical difficulty of removing beads prior to flow cytometry analysis. Irrespective of CD32b expression, differences in the functional effects IgG-IC CD32b inhibition were most notable in the IgM^hi^ MZ-like B cell subpopulation, particularly in patients with CIS or MS. Therefore, it may be that CD32b expression and/or functional effects of CD32b engagement and activation of the downstream signalling pathway is of most significance in the IgM^hi^ MZ-like B cells. Alternative pathways of regulation may be more important to investigate in other B cell subpopulations.

BAFF levels in serum negatively correlated with CD32b expression on naive and IgM^lo^ MZ-like B cells, as well as total serum IgM levels. Serum IgM levels were significantly higher in females, as has been previously reported (58), and therefore the negative correlation of total IgM with naive B cell CD32b may have been confounded by the sex difference in CD32b expression. However, our finding that serum BAFF was negatively correlated with both naive B cell and IgM^lo^ MZ B cells CD32b expression is consistent with an ability of murine BAFF to inhibit CD32b expression in CpG-activated B cells (59) and evidence that BAFF promotes IgM but not IgG secretion in cultured memory B cells (60). Although BAFF was not measured in the controls in this study, levels in serum from healthy individuals are generally reported to be lower than in MS serum (42). BAFF is a cell survival and maturation factor for B cells (61) and may increase cell migration in response to CXCL13, which is elevated in the cerebrospinal fluid of people with CIS and MS (62, 63). BAFF expression is downregulated by testosterone and upregulated by oestradiol in mice (64, 65), and although the difference was not statistically significant, there was a trend towards lower serum BAFF levels in males compared with females in this study. Downregulation of the expression of inhibitory CD32b on B cells of patients with rheumatoid arthritis can be a regulatory response to increased B cell activating stimuli, such as CD40 engagement on MBC (26). Furthermore, overexpression of BAFF in humans due to genetic polymorphisms is associated with increased risk of MS and other autoimmune diseases (66), though we did not examine our cohort for these. In teleost fish and mice, BAFF upregulates MHC II (67, 68), which may increase B cell-mediated antigen presentation to T cells (69) and be important for promoting self-reactive T cell activation in MS. These findings suggest that the lower CD32b expression in cells from females with CIS or MS may be a consequence of increased serum levels of BAFF or other B cell activating stimuli associated with MS, and raise the possibility that CD32b is downregulated *via* a negative feedback mechanism downstream of CD32b following B cell activation. Notably, Atacicept, an IgG_1_ Fc region fusion protein that binds BAFF and a proliferation-inducing ligand (APRIL) resulted in higher rates of MS relapse and conversion to MS in clinical trials (70, 71). Although the mechanism for more relapses with Atacicept has not been elucidated, it has the capacity to deplete potential B regulatory cells (B_regs_) in the pre-memory B cell compartment and cause transient increases in potentially pathogenic CD27^+^ B cells (72, 73). In addition, Atacicept decreases serum Ig levels, potentially reducing their interactions with inhibitory B cell Ig receptors such as CD32b (74).

Given that seven of the females in this study with CIS or MS had IgM antibody responses to EBV, and EBV latent infection can upregulate BAFF secretion in infected B cells (75), it is tempting to speculate that EBV reactivation could be the trigger for increased serum BAFF and lower expression of CD32b on some B cell subsets in people at the earliest stages of MS. Low but detectable levels of anti-EBV IgM antibodies alongside anti-EBV IgG antibodies probably indicate EBV reactivation, which can be caused by other viral infections (76, 77). Without further testing, for example with heterophile antibody assays that may exclude EBV reactivation (78), the EBV reactivation status of these individuals is unclear. The proportion of B cells that were EBV-infected was not measured, and although EBV may infect MZ-like B cells, it is more likely to reside in class switched MBC (79). However, the finding that anti-EBV IgM antibodies were detected primarily in CIS patients suggests that EBV reactivation could be a precipitating factor in the first but not subsequent demyelinating episodes, though this would need to be confirmed in a larger study.

Incubation of PBMC with R848-induced IL-10 expression in naive, transitional and IgM^+^ DN B cells, and IL-10 expression was inhibited by IgG-IC, but the effect was independent of CD32b. Addition of CD32b-blocking BI-1206 antibody, rather than increasing IL-10 expression, further decreased IL-10^+^ cell frequencies in these cell types, but the cause of this observation is unclear. Nevertheless, both the pattern of higher CD32b expression on IgM^+^ B cells, and increased frequencies of IL-10^+^ cells in these subsets provided further evidence that B_regs_ are enriched amongst IgM^+^ MBC, which include MZ-like B cells (80). Although far less abundant than naive B cells, which are another reported B_reg_ reservoir, MZ-like B cells were in similar abundance to transitional B cells, which also contain many IL-10 expressing cells (80).

In summary, females with CIS or MS had decreased CD32b expression on naive and IgM^hi^ MZ-like B cells compared with female controls, which was associated with increased serum BAFF and IgM, and seropositivity for EBV VCA IgM antibodies. In controls, CD32b expression on naive and IgM^hi^ MZ-like B cell subsets was required for IgG-IC-mediated inhibition of TNF production by polyclonally activated cells, which was demonstrated by CD32b blockade experiments. However, no consequence of the lower CD32b expression on B cells from CIS or MS patients was detected using this approach. Naive and MZ-like B cells are “early responders” to infections and endowed with regulatory activity, and are both affected by successful MS therapies (81). Therefore, further investigations of the role of differential CD32b expression on these B cell subpopulations in the context of EBV infection and MS immunopathogenesis are warranted, particularly their roles in activation and/or regulation of T cell responses.

## Data Availability Statement

The raw data supporting the conclusions of this article will be made available by the authors, without undue reservation.

## Ethics Statement

The studies involving human participants were reviewed and approved by the Sir Charles Gairdner Hospital Human Research Ethics committee (2006-073) and Bellberry Human Research Ethics Committee (2014-02-083). The patients/participants provided their written informed consent to participate in this study.

## Author Contributions

ST, PH, MF, AK, IT, and BF designed the study. ST, JL, MF, and PH formulated analyses. ST conducted experiments and data analysis. ST wrote the first draft of the manuscript. ST, JL, IT, BF, AK, MF, and PH were involved in revising the manuscript and interpreting the findings. All authors contributed to the article and approved the submitted version.

## Funding

This work was supported by Multiple Sclerosis WA and a project grant from Multiple Sclerosis Research Australia (ID 19-0658). CIS participants were recruited during a trial supported by the National Health and Medical Research Council of Australia (ID 1067209).

## Conflict of Interest

IT and BF were employed by BioInvent International AB.

The remaining authors declare that the research was conducted in the absence of any commercial or financial relationships that could be construed as a potential conflict of interest.
