# Supplementary material for: FcγRIIb Expression Is Decreased on Naive and Marginal Zone-Like B Cells From Females With Multiple Sclerosis
Source: Front Immunol. 2021 Jan 11;11:614492. doi: 10.3389/fimmu.2020.614492 (PMC7832177; doi:10.3389/fimmu.2020.614492)
Supplement: Supplementary file 1 [file Table_1.docx]

Supplementary Material

# Supplementary Figures and Tables

Supplementary Table 1. Characteristics of the healthy controls (HC), patients with clinically isolated syndrome (CIS), and patients with multiple sclerosis (MS) in the cohort.

|  | Healthy controls (n=16) | CIS (n=14) | MS  (n=8) | p-value |
| --- | --- | --- | --- | --- |
| Female sex [n, (%)] | 9 (56%) | 8 (57%) | 5 (63%) | p=0.96 |
| Age [median, (IQR)] | 41.5 (33.0–47.6) | 36.1 (29.0–40.8) | 45.0 (35.7–46.4) | p=0.31 |

*The comparison of the equality of distribution of female sex between groups was made using a Chi-squared test. The comparison of age between controls and patients with CIS and MS was performed using a Kruskal-Wallis test.*


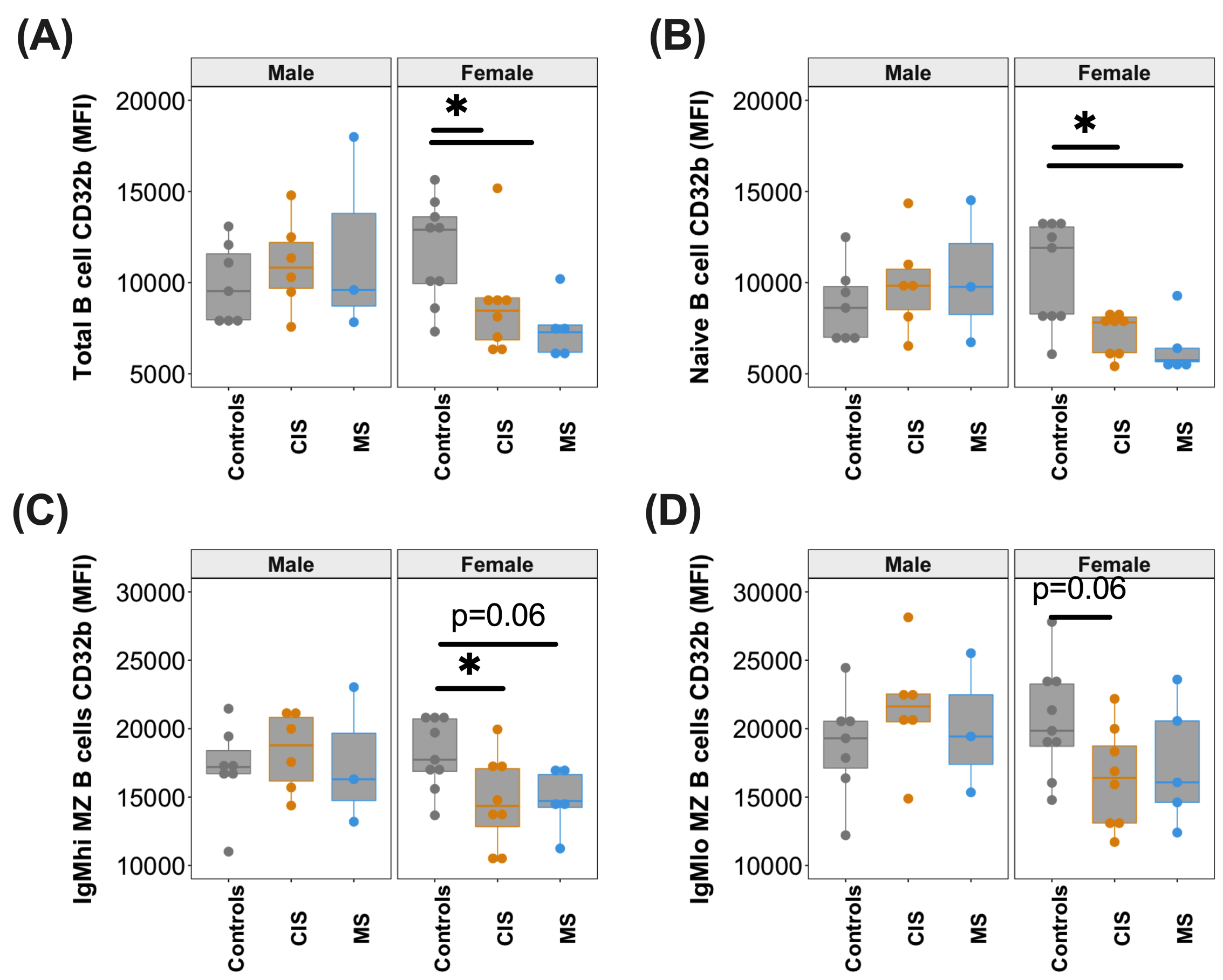


Supplementary Figure 1. CD32b expression on B cell subsets from males and females with CIS and MS compared with controls. CD32b expression is shown using Tukey’s boxplots, with median and interquartile range shown, for (A) total B cells, (B) naive B cells, (C) IgM^hi^ MZ B cells, and (D) IgM^lo^ MZ B cells from healthy controls (n=7 males, 9 females; grey circles), and patients with CIS (n=6 males and 8 females; orange circles) or MS (n=3 males and 5 females; blue circles). A dot is shown for each individual. P-values shown are derived Mann-Whitney tests; *p<0.05.


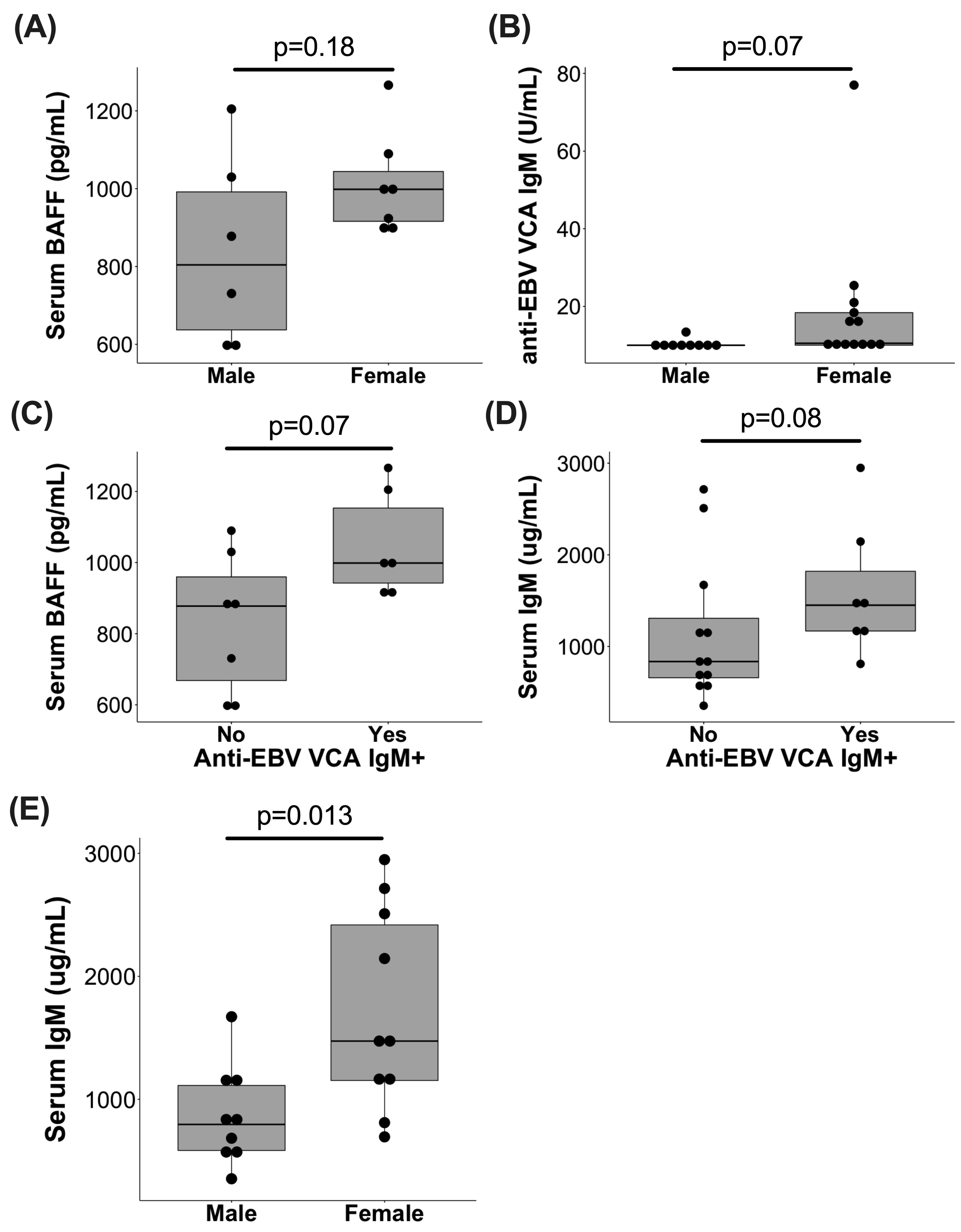


Supplementary Figure 2. The relationships between serum B cell activating factor (BAFF), sex, and anti-Epstein Barr Virus (EBV) Viral Capsid antibody (VCA) IgM antibodies in sera from patients with CIS or MS. Data are shown as Tukey’s boxplots and figures show (A) serum BAFF levels in females with CIS or MS (n=7) and males (n=6) with CIS or MS; (B) anti-EBV VCA IgM levels in females (n=13) and males (n=9) with CIS or MS; (C) serum BAFF levels in patients with CIS or MS with anti-EBV VCA IgM antibodies detected in sera (n=6) compared with those that were seronegative (n=7); (D) total serum IgM in patients with anti-EBV VCA IgM antibodies present in sera (n=7) compared with seronegative patients with CIS or MS (n=12); and (E) total serum IgM in females with CIS or MS (n=10) compared with males with CIS or MS (n=9) . A dot is shown for each individual. Groups were compared using Mann-Whitney tests.

**
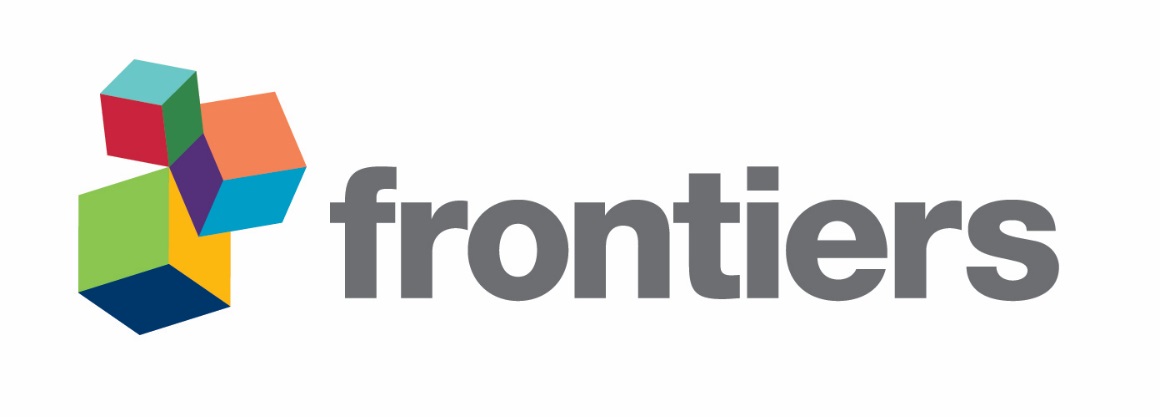
**
